# Emotional and Psychological Safety in Healthcare Digitalization: A Design Ethnographic Study

**DOI:** 10.3389/ijph.2024.1607575

**Published:** 2024-09-06

**Authors:** Mara Vöcking, Anne Karrenbrock, Andreas Beckmann, Carmen Vondeberg, Laura Obert, Bernhard Hemming, Peter Minartz, Christian Bleck, Diana Cürlis, Silke Kuske

**Affiliations:** ^1^ Fliedner Fachhochschule Düsseldorf, Düsseldorf, Germany; ^2^ Department of Social and Cultural Sciences, Hochschule Düsseldorf, University of Applied Sciences Düsseldorf, Düsseldorf, Germany; ^3^ Department Münster School of Design, FH Münster, University of Applied Sciences, Münster, Germany

**Keywords:** emotional safety, psychological safety, digital technology, healthcare, participatory research

## Abstract

**Objectives:**

Emotional and psychological safety is important during the use of digital technologies in healthcare. We aimed to gain comprehensive insight into needs, influencing factors and outcomes in the context of perceived safety and digital technologies in healthcare.

**Methods:**

We employed a participatory, design ethnographic research approach with 16 participants in 10 use cases. The methods included in an iterative process were, think-aloud, guideline-based interviews, process mapping, storyboard creation, and photo documentation. A qualitative, primarily inductive data analysis and synthesis was performed.

**Results:**

Perceived safety is influenced by various factors and unmet needs. Increased perceived safety can positively support the use of digital technologies, whereas low perceived safety can limit or even hinder its use.

**Conclusion:**

The needs of the different target groups should be considered throughout the entire process of digital technology development and healthcare provision to support their implementation. These findings support further research by providing specific aspects of emotional and psychological safety regarding target groups, settings, and ages and those with different levels of affinity for digital technologies.

## Introduction

Digital transformation, as a response to COVID-19 [1], affects many areas of society [2] and is related to perceived safety in healthcare [1]. The implementation of digital technologies (DTs) provides new opportunities, e.g., by strengthening the empowerment of healthcare recipients (HCRs) [3] and supporting flexible care provision through analog and digital care by healthcare providers (HCPs), e.g., through telemedicine [4]. However, several studies have shown concerns regarding safety and security related to DTs [1] and have recently stressed the need to investigate emotional (ES) and psychological safety (PS) related to patient safety [5, 6]. A differentiation between “feeling safe” and “being safe” in healthcare is required. The consequences of not feeling safe can include the loss of trust, fear, trauma, and in the further course restricted healthcare use [5]. From a public health point of view, how people perceive risks, related emotions, efficacy, information or trust perceptions, can also be associated with sense of public health safety [7].

In general, ES is related to a feeling that is located on a continuum between feeling safe and feeling threatened and that is influenced by internal and external conditions and factors [8] and PS is defined as perceived safety in the context of the work environment and team dynamics [9].

Although, several studies considered perceived safety related to DT, only a few have studies investigated it as a primary research focus in the recent years. These mainly qualitative studies were restricted to a limited target group, such as older people or selected DTs such as robotics, assistive technology or telecare [8]. For example, Akalin et al. stressed in a “two-by-five mixed-subjects design experiment” of a human-robot interaction (N = 27) that perceived safety is a key factor in sustaining interaction, collaboration, and acceptance in the context of the use of DTs in healthcare and is related to a sense of control, trust, and comfort [10]. Others have reported that DT use in a simple design can enhance perceived safety for elderly people [11] and that a robot design can decrease perceived safety by either looking too human-like or not having enough human traits [12]. Understanding and prioritizing the needs of DT users can support their acceptance of DTs [13]. In summary, although the phenomenon is relevant in healthcare, currently, the evidence is limited. This is especially true for the psychological safety of DTs use [8]. Therefore, we aimed to gain deeper insight into needs, influencing factors, and outcomes in the context of emotional and psychological safety and DT in healthcare.

## Methods

### Design

Our design ethnographic approach (DEA) [14] involves participants as co-designers, considering scientific standards [15] and the “involvement” level of participation [16] to improve evidence by participatory methods [17]. DEA is “(…) interpretative, qualitative, engaged, active, constructivistic, interactionistic, phenomenological, explorative, and abductive.” [14] Usually, case studies are applied, that include one or more cases to investigate poorly researched “real-world phenomena in complex contexts” across various settings [18, 19]. We also provided a real-world user experience [20] to uncover users’ needs and feelings to investigate an even deeper level of user expression, by addressing and observing what individuals say, do, and create [21]. Insights about implicit and tacit knowledge (and needs) could be gained, e.g., skills that people are capable of but that are not easily articulated verbally [22]. Member checking [23] was conducted. This study was conducted as part of the research project titled “Emotional safety as a condition for success of the digital transformation in healthcare (SteTiG),” registered at the Open Science Framework: https://doi.org/10.17605/OSF.IO/UTSQN. Our study was approved by the ethical committee of Fliedner Fachhochschule Düsseldorf: 04/2022. Ethical advice from the Ethical Committee Ärztekammer Nordrhein: 2022107.

### Sample Design and Setting

Criterion-based convenience sampling [24] was performed, which resulted in 16 participants in 10 design ethnographic (DE) use cases. We recruited participants on the basis of expert and project member recommendations as well as snowballing. HCRs of different ages (e.g., children, adults, and elderly individuals), genders, and settings were included. Different disease patterns were considered (e.g., people with acute and/or chronic diseases). HCPs, such as physicians, psychotherapists, paramedics, and nurses were considered. A family member also took part in the study to support an underage child and to add a family perspective. The sampling of heterogeneous use cases was primarily based on the WHO classification of digital health interventions [25] which allowed us to observe differences in perceived safety with respect to DTs (see Table 1): a personal health tracking (1) diet app and (2) a sleep app; (3) a mobile electrocardiogram; (4) a closed-loop system for diabetes type 1; (5) virtual reality (VR) and (6) robotics in care facilities; (7) hospital information system; (8) telemedicine psychotherapy; (9) tele-psychotherapy; and (10) simulation training in emergency care.

**TABLE 1:** World Health Organization classification of digital health interventions-based description of use cases (North Rhine-Westphalia, Germany. 2023).

World Health Organization classification of digital health interventions		1. Interventions for clients						2. Interventions for healthcare providers			
		1.4 Personal health tracking				1.8 Lifestyle intervention tools (Hermann et al. [26])		2.1 Client identification and registration	2.4 Telemedicine		2.8 Healthcare provider training
		1.4.2 Self-monitoring of health or diagnostic data by client		1.4.3 Active data capture/documentation by client		1.8.1 Digital psychosocial facilitation		2.1.2 Enroll client for health services/clinical care plan	2.4.1 Consultations between remote client and healthcare provider		2.8.1 Provide training content to healthcare provider(s)
Use cases		1	2	3	4	5	6	7	8	9	10
Digital technology		Diet app, used on smartphone	Sleep app, used on laptop	Mobile electrocardiogram	Closed-loop system pump	Virtual tours via Google Earth using virtual reality glasses	Robot	Electronic health record in hospital information system	Telemedicine psychotherapy via video consultation tool	Tele-psychotherapy via video consultation tool**	Simulation training in emergency care with electrocardiogram patient simulator
Participants*		HCR, patient with obesity	HCR, patient with insomnia; HCP, doctor	HCR, patient with AVNRT	HCR 1, child, patient with diabetes type 1; HCR 2, family member	HCR, elderly person in retirement home; HCP 1 and 2, nurses	HCR 1 and 2, elderly people in retirement home; HCP, nursing home project manager	HCP, internist	HCR, patient with psychosis	HCP, child and adolescent psychotherapist	HCP, instructor/paramedic
General setting		Germany									
Setting in daily live		Usually used at home, after a meal or in the evening	Usually used at home, before or after sleep	Everywhere, especially at home	Everywhere	Retirement home, lounge area	Retirement home, lounge area	Hospital, doctors’ room	At home, living room	Doctors’ office/At home, living room	Fire and rescue service academy, simulation room
Selected research setting	1. Visit°	At home, living room table^a^	Doctors’ office, patient room^b^	At home, living room^a^	Bakery^b^	Retirement home, lounge area^b^	Retirement home, lounge area^b^	Hospital, doctors’ room^a^	At home, living room^a^	At home, living room^a^	Fire and rescue service academy, simulation room^a^
	2. Visit°°	At home, living room table^a^	Doctors’ office, patient room^a^	At home, living room^a^	Bakery^b^	Retirement home, lounge area^a^	Retirement home, lounge area^b^	Zoom^a^	At home, living room^a^	At home, living room^a^	Zoom^a^
	3. Visit°	At home, living room table^a^	Doctors’ office, patient room^b^	At home, living room^a^	Bakery^b^	Retirement home, lounge area^a^	Retirement home, lounge area^b^	Zoom^a^	At home, living room^a^	Zoom^a^	Zoom^a^
Data collection		Researcher 1				Researcher 2	Researcher 1		Researcher 2	Researcher 1	

Legend: * = for more details about participants see Table 2: participants’ characteristics; ** = digital technology only used during COVID-19 lockdown; interview time: ° = 30 min single participant, 45–60 min for more than one participant; °° = 45 min single participant, 60–90 min for more than one participant.

HCR, healthcare recipients; HCP, healthcare provider; AVNRT, atrioventricular nodal reentrant tachycardia; researcher 1 and 2 = social and product designers.

^a^ One-to-one interview.

^b^ Group-interview.

This study was conducted in Germany, toward the end of the COVID-19 pandemic from July 2022 to February 2023. DTs have become more important in several areas of life and public communication [27]. Therefore, diverse familiar real-world settings were considered (*in situ* and online) to capture the full spectrum of DT usage and to ensure that participants felt at ease. Related disturbances, e.g., people passing by, were accepted as authentic parts of the real-world context.

### Data Collection

**FIGURE 1:**
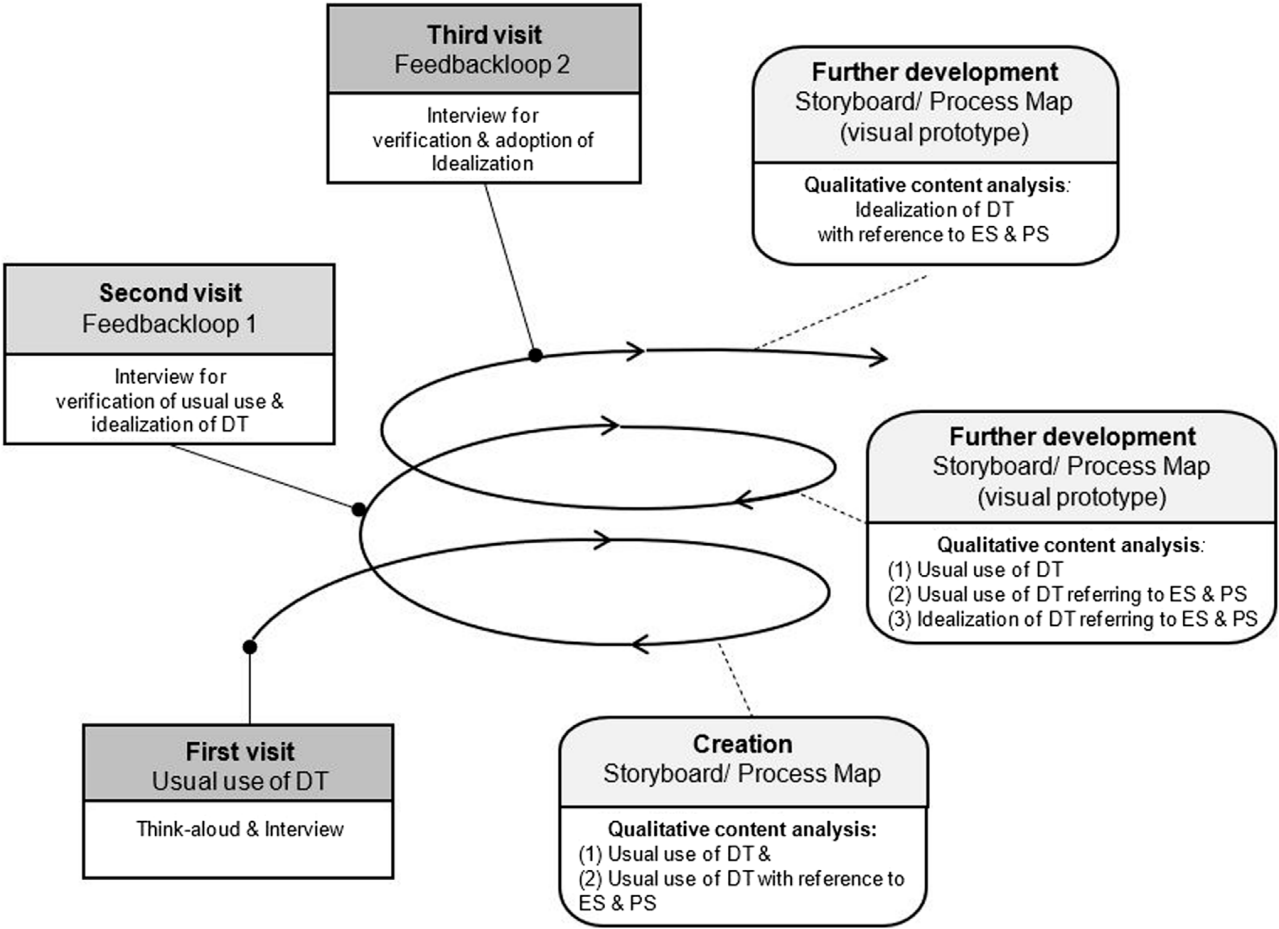
Design ethnography approach—definitions, data collection and analysis (North Rhine-Westphalia, Germany. 2023). Legend: ES, emotional safety; PS, psychological safety; DT, digital technology. Definitions of methods and techniques in data collection and analysis: 1) Think-aloud technique [28] = it aims to collect data about a cognitive process by verbalization and working memory regarding subject and task. 2) Guided semi structured interviews [29] = semi structured interviews contain “(…) prepared questioning guided by identified themes in a consistent and systematic manner interposed with probes designed to elicit more elaborate responses (…) to help direct the conversation toward the topics and issues about which the interviewers want to learn”. 3) Storyboarding [30] = “Storyboarding is the process of describing a user’s interaction with the system over time through a series of graphical depictions and units of textual narrative” 4) Process mapping [31] = it refers encompassing understanding of the process and contains “(…) identification, information gathering, map generation, process analysis and taking improvement forward.” 5) Structure formation technique (SFT) [32] = in core, it “(…) consists in passing on a system of rules which allow for visualizing the structure of each particular subjective theory (…) to make the dialogue-consensus between research subject and research object possible (…) according to the dialogue consensus criterion of truth (…) to approximate an ideal speech situation as closely as possible.”

For each use case three visits (see Figure 1) were performed using the think-aloud technique [28], guided semi structured interviews [29], storyboards [30] supplemented with a process map [31], and a structure formation technique (SFT) [32] for data collection. The process was documented using audio-records, photographs, field notes and observation protocols.

The first visit was performed *in situ* to better involve vulnerable groups and online Zoom visits were conducted upon participant request. Zoom interviews followed the same structure by using a digital visualization tool (Miro boards). Sociodemographic data, health status, self-reported technical affinity and the specific type of DT were recorded beforehand.

For each data collection phase an interview guide [29] (see Supplementary Material 1) was developed. The first guide involved a think-aloud approach and the user was encouraged to share feelings and thoughts while using the DT. Then associations of perceived safety were discussed. Finally, a closing question was posed to provide the opportunity for additional information or thoughts. The second guide included a member check of the findings of the usual use of the DT from the first phase, an idealization of the user experience design of the DT in relation to perceived safety, and the same closing question from the first visit. A process map was provided to the participants to reflect on the usual DT use and to ask for corrections, if needed. For the idealization of DT usage and its design, an imaginary space was opened where all ideas were allowed. The third guide included a member check of the idealized DT use and its design, as well as an opportunity to make additions. To check for completeness and correctness, the visualizations of the ideas were translated into storyboard process maps and visual prototypes. The interviews with the underaged child were conducted using shorter and simpler language. The child’s sister was present and took part on her own request.

### Data Analysis and Synthesis

A qualitative inductive content analysis was performed [33] by four researchers (MV, AB, CV, and LO). Peer group sessions and supervision (SK) were performed. A final harmonization of the terminology and clustering of domains on the basis of the core dimensions was performed by one researcher (MV). First, the data for each use case were analyzed with respect to the influencing factors, needs, and outcomes that would serve the design of future DTs in the healthcare sector, taking perceived safety into account. General thoughts and feelings about usual DT use were analyzed separately from the results concerning perceived safety to determine aspects that went beyond the context of feeling safe. Second, the core dimensions, main categories, and subcategories were developed. The synthesis [34] considered the different DTs, HCRs and HCPs related to ES and PS, and digital affinity. Complementary or specific categories are presented separately. Finally, content related domains were developed.

## Results

### Participants

A total of 30 visits were conducted across the 10 use cases. Sixteen participants were included in the 10 selected use cases (see Table 2). The age ranged from 11 to 86 years, and 12 out of the 16 participants were women. Participants with chronic or acute disease were recruited, with some having only a chronic or acute disease. According to our defined target groups, nine HCRs and seven HCPs were included.

**TABLE 2:** Participants’ characteristics (North Rhine-Westphalia, Germany. 2023).

Use cases/digital technology		Total n	Diet app	Sleep app	Mobile electrocardiogram	Closed-loop system	Virtual Reality	Robotic	Hospital information system	Telemedicine psychotherapy	Tele-psychotherapy for children	Simulation training in emergency care
Total number of participants*		16	1	2	1	2	3	3	1	1	1	1
Project collaborators included		1	—	1	—	—	—	—	—	—	—	—
Gender	Female	12	1	1	1	2	2	2	—	1	1	1
	Male	4	—	1	—	-	1	1	1	—	—	—
	Various	—	—	—	—	—	—	—	—	—	—	—
Age	in years RA ± SD	11–86 45,56 ± 24,2	53	61–63 62 ± 1	29	11–23 17 ± 6	19–86 43.67 ± 30.07	29–86 66.33 ± 26.41	60	33	32	34
Final school degree	Lower-level degree	3	—	—	—	—	1	2	—	—	—	—
	Average-level degree	1	—	1	—	—	—	—	—	—	—	—
	Higher-level degree	11	1	1	1	1	2	1	1	1	1	1
	Other (e.g., primary school diploma)	1	—		—	1	—	—	—	—	—	—
Education (multiple response)	Vocational (school or academy)	4	1	1	—	—	—	2	—	—	—	—
	University or college degree	9	—	1	1	1	1	1	1	1	1	1
	Missing value (no data available)	3	—	—	—	1	2	—	—	—	—	—
Other education	e.g., specialized doctor status, license to practice	2	—	—	—	—	—	—	1	—	1	—
Target group roles	Healthcare recipients	9	1	1	1	2	1	2	—	1	—	—
	Of this group, family members	1	—	—	—	1	—	—	—	—	—	—
	Healthcare providers	7	—	1 (a)	—	—	2 (b)	1 (c)	1 (a)	—	1 (d)	1 (e)
Disease	Chronic disease	3	—	—	—	1	1	—	—	1	—	—
	Acute disease	1	—	—	1	—	—	—	—	—	—	—
	Chronic and Acute disease	4	1	1	—	—	—	2	—	—	—	—
	Neither	8	—	1	—	1	2	1	1	—	1	1
Technical affinity (f)	High tech-savvy	10	1	1	1	2	2	1	—	1	—	1
	Moderately tech-savvy	2	—	1	—	—	—	—	—	—	1	—
	Low tech-savvy	4	—	—	—	—	1	2	1	—	—	—

n, number of participants; R, range; A, average; SD, standard deviation; *all participants are German.

(a) Doctor (b) Nurse (c) Nursing home project manager (d) Psychotherapist (e) Paramedic (f) Self-reported technical affinity was recorded before the interviews.

### Impact of Perceived Safety

Perceived safety has influenced all the target groups’ DT usage behavior, thoughts, emotions, and needs. In total, 13 outcomes (see Supplementary Material 2A) were associated with low or strong perceived safety in different contexts and with different DTs. We observed that a low level of perceived safety had an influence on the implementation of DTs: use depending on certain circumstances (n = 6), nonuse (n = 4), partial use (n = 3), or use other than intended (n = 1). In the case of DT use, depending on certain circumstances, the HCPs weighed the benefits of a DT according to their patients’ health status and one HCR was afraid to use the DT autonomously because of fears of breaking something or doing something wrong. The non-use of DTs was related to limited competencies, control, discomfort, and increased risk perception. Partial use was related to feelings of danger, limited functionalities, limited control, competencies, and knowledge. In one case, because of limited perceived safety, the DT was used in another way than intended due to its limited functionality. In six cases, low perceived safety had an impact on HCPs’ and HCRs’ thoughts and emotions, including skepticism, mistrust, and discomfort. In contrast, strong perceived safety had, in four use cases, a positive influence on DT use and thus its implementation. For HCRs, regular DT use was related to aspects of trust, recommendations, and positive health effects. In general, the use of DTs was not classified as risky.

### Influencing Factors and Needs

A total of 13 domains were developed on the basis 40 core dimensions containing ES and PS aspects. Thereof 22 core dimensions that cover influencing factors (CDIFs) and needs (CDNs). Fourteen core dimensions were exclusively addressed by influencing factors and four core dimensions by needs. The CDIFs/CDNs were based on 150/48 main categories and 232/90 subcategories identified from the 10 use cases (see Supplementary Material 2B–D).

**FIGURE 2:**
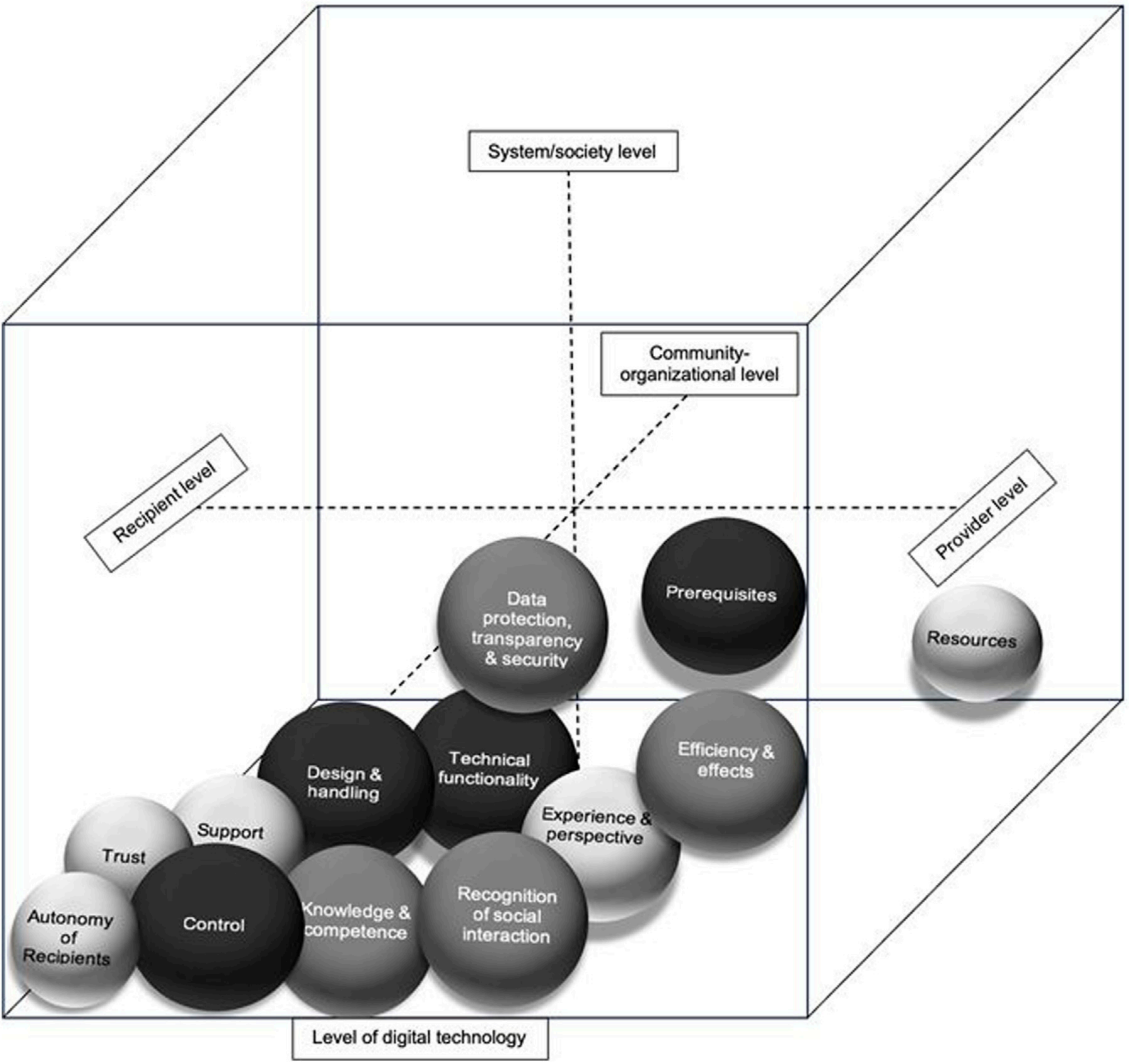
Core dimensions of influencing factors and needs in the context of feeling safe and digital technologies in healthcare (North Rhine-Westphalia, Germany. 2023). Legend: larger black spheres = mostly addressed CDIFs/CDNs; smaller lighter spheres = rarely addressed CDIFs/CDNs; bottom layer = digital technology level; left layer = recipient level; right layer = provider level; upper layer = system/society level; and rear layer = community-organizational level.

The domains covered four levels: the DT level, the individual level, the community-organizational level, and the system-society level. The participants focused mainly on the individual level, particularly in relation to DTs (see Figure 2). Among the 13 domains, nine were addressed by both the HCRs and HCPs, three were addressed primarily by the HCRs, and one was addressed only by the HCPs.

### Perspective of Healthcare Recipients and Providers

Most of the CDIFs and CDNs were addressed in several use cases and were covered by all target groups related to ES and PS. The four domains “*design and handling*,” “*technical functionality*,” “*control*” *and* “*prerequisites*” were addressed in all ten use cases. While the domain “*control*” focused mainly on the DT level, “*design and handling*” and “*technical functionality*” additionally took the community-organizational level into account. “*Prerequisites*” took all levels into account. All four domains considered ES and PS.

The “*design and handling*” addressed in terms of ES and PS aspects included two related topics: user-friendliness, which was mentioned most frequently, and the flexible availability and usability of DTs, which were covered by needs. The need for DT optimization was also mentioned frequently, predominantly by those with a high level of DT affinity.

“It’s a great program, and it’s easy to use. (…) I feel safe using it because it's simple. (…)” (HCP, tele-psychotherapie, 1st visit)

“*Technical functionality*” was related to available and reliable digital health data transfer for HCPs to ensure the quality and safety of a correct diagnosis and treatment. The need to improve the PS was related to the availability of patient data via various DTs, including the HCR’s health data/feedback to the HCP. Furthermore, the PS was related to the technical reliability of DTs and was also mentioned as a need. In three cases, the autonomous, reliable function of DTs was mentioned. For one HCP, it was important to differentiate between technical errors and one’s own mistakes related to the DT.

“But I always find it difficult when it’s caused by the technology. And the participants can’t understand that it’s a technical problem right now (…). That’s actually what I (…) mean by perceived safety (…)” (HCP, simulation training in emergency care; 3rd visit)

The domain “*control*” encompassed three types of control: gaining control through the DT, being in control of the DT, and (gaining) control over the DT through analog backup/redundant measures. Gaining control through the DT, especially over one’s own health status, helped the participants feel protected and independent, which led to ES. This CDIF was exclusively mentioned by HCRs, who used DTs for personal health tracking independently from an HCP. Being in control of the DT was mentioned frequently and included aspects with a negative influence on perceived safety, such as a lack of control (over HCRs), to ensure the integrity of the HCR and the DT. (Gaining) control over of the DT through analog backup/redundant measures was mentioned by a child with diabetes. These backup measures, such as a glucometer or spare batteries, could provide supportive ES in the case of (possible) DT failure. The patient, a child, also described her parents’ control as a guarantee influencing ES. The patient’s sister mentioned the DT (diabetes pump) itself as a strong factor for ES, as it took control through autonomous functions.

“I can tell you how our parents feel about it: very, very safe. They know for a fact that when she’s at school and has the pump, it’s super safe (…)” (Family member, closed-loop system; 1st visit)

The domain “*prerequisites*” addressed the CDIF related to usage and implementation concerns associated with the DT, which was addressed in seven cases for ES and PS. These concerns reflected aspects such as legal restrictions on the choice of the DT. This domain also included the aspects of freedom from pain while using DTs and eliminating hazards.

“(…) the risk of accidentally defibrillating yourself is simply too high. That would of course be a major safety hazard in terms of perceived safety. That can’t actually happen here (with the DT) (…).” (HCP, simulation training in emergency care, 1st visit)

The way in which DTs are used, e.g., certainty about the physical wellbeing of HCRs and HCPs during the use of DTs, plays a role in perceived safety. In this context, the design and usage of DTs seemed to be particularly relevant because they pose a potential physical safety risk. In particular, the HCPs expressed the need for physical safety when DTs were used to support ES and PS.

The domain “*knowledge and competence*” addressed in nearly all of the cases (n = 9) encompassed two interdependent frequently mentioned CDIFs at the DT level: recipients’ knowledge and competence toward the DT and familiarity based on regularity of use. These CDIFs reflect the perceived familiarity of the user, who develops knowledge, competences, and self-confidence through repeated or regular use, which supports ES and PS.

“The content (of the app) gives me a sense of safety, and I learn a new what I have already read (*…*)” (HCR, sleep app, 2nd visit)

The latter CDIF was the most frequently mentioned by both the HCPs and HCRs. The need to develop a habit through the regular use of and early introduction to DTs was mentioned by the HCRs and HCPs of the robotic and VR technology use cases. Another CDIF that was frequently mentioned concerned the HCRs’ knowledge of and competence in DTs and had a strong influence on ES. The CDIF related to self-confidence in DT use was mentioned mostly by vulnerable groups (the child and elderly individuals). This CDIF was strongly related to support during DT use, as mentioned by the HCRs. In the case of a lack of self-confidence, support in dealing with DTs independently was needed. Additionally, support and guidance are needed during DT use to improve ES.

The domain “*efficiency and effects*” (n = 8 cases) contained mostly PS, e.g., efficient healthcare provided by the DT as well as the health and care effects associated with the DT. This domain was mentioned by both the HCPs and HCRs.

“(…) I would actually like to be informed when I order something and it's done. (…) It would be much better for my safety and also for patient safety (…)” (HCP, hospital information system, 1st visit)

The accessibility of DTs was mentioned in the context of VR. The domain has been discussed much at the DT level, but some factors have also been addressed at the organizational level.

The domain “*data protection, transparency and security*” (n = 8 cases) was nearly evenly relevant to both ES and PS, expressed at all levels. The CDIF concerning secure data management and protection was addressed at the DT, organizational and system levels. The transparency of DT data management was a concern at the technology-organizational level, especially for the HCPs. The domain “*recognition of social interaction*” was also covered by 8 cases located at the DT level and included the exchange of experience with DTs between HCRs and HCPs, which enhanced perceived safety. A reduction or loss of familiar interpersonal interactions/relationships, e.g., between HCPs and HCRs, was related to low ES in the case of unmet needs. The opportunity for visual interaction independently of DTs was mentioned by the HCPs as a need to promote the doctor‒patient relationship to promote feelings of safety for both parties. Inadequate healthcare because of limited interpersonal interaction due to the presence of DTs was mentioned by both the HCRs and HCPs using teletherapy, resulting in a low perceived safety.

The “*support*” domain (n = 7 cases) was almost exclusively mentioned by the HCRs. Support in the context of DTs was mentioned as a core dimension of the factors influencing ES and needs related to ES. The effective inclusion of medical expertise using DTs was related mostly to the technology level, whereas support was seen partly at the organizational level. The inclusion of medical expertise through DTs was discussed in cases where HCRs tracked their own health to increase perceived safety. The “*experience and perspective*” domain, which was represented in seven cases, addressed CDIFs related to ES and PS equally. The need for humans in healthcare and their irreplaceability by DTs was mentioned by an HCP in the robotic case. (Negative) feelings during DT use inhibited perceived ES, especially for vulnerable users.

The domain “*trust*” (n = 6 cases) was covered by only one CDIF at the DT level. The factor related to trust in and by HCPs was addressed by both HCRs and HCPs as relevant for ES. HCPs were seen as a mediator of trust in DTs. As HCP act as reference persons, their emphatic and credible interactions and the transfer of knowledge build trust. We observed that trust in human beings was greater than that in DTs and supported ES. The promotion of trust was also mentioned by the HCPs as a CDIF and CDN for successful implementation. The maintenance of trust in DTs was stressed, as was the presupposed relationship for further DT use.

The domains “*resources*” (n = 5 cases) and “*autonomy of recipients”* (n = 3 cases) were covered by only some CDIFs. Resource-efficient healthcare via DTs could lead to increased PS; in contrast, challenges and concerns about the effort and time involved in using DTs could hinder PS. A feeling of sovereignty and empowerment was gained through DT use, leading to ES.

### Digital Technology and Target Group Related Perceived Safety

Across all of the technologies, the domains “design and handling,” “control” and “technical functionality” were addressed in terms of feelings of safety. “Autonomy of recipients” was mentioned only in relation to apps, mobile electrocardiograms and video consultation tools. “Data protection, transparency and security” is mentioned in relation to apps, mobile electrocardiograms, robotics, hospital information system, video consultation tools and electrocardiogram patient simulation equipment.

We observed that various target groups held distinct perspectives on perceived safety. Most of the target groups covered many different domains. People with high to moderate levels of affinity for technology addressed domains such as “*design and handling*,” “*efficiency and effects*,” “*knowledge and competence*,” and “*prerequisites*” with the latter involving usage and implementation concerns related to DTs. HCPs with a lower level of affinity for technology focused mainly on the reliability of digital health data.

Elderly participants expressed a strong need for an introduction to DTs by familiar and trusted persons for perceived safety. Human factors and involvement can significantly influence ES. HCPs, informal caregivers, and peer support all contribute to ensuring that vulnerable HCRs feel emotionally safe when adopting and using DTs. However, uncertainty in the independent use of DTs without support and uncertainty regarding the correct use and termination of DTs had negative effects on feelings of safety. In contrast, existing competences in relation to DTs had a positive impact on perceived safety among HCRs with low technical affinity.

## Discussion

This study provides multifaceted insight into the various participants’ experiences of ES and PS, perspectives, and needs, providing valuable insights into improving the use, design, and implementation of DTs in healthcare contexts, captured by 13 domains. Akalin et al. [10] described six domains of perceived safety in the case of social human–robot interaction based on subjective and objective measures that were similar to ours: “*control*,” “*trust*,” “*experience*” and “*transparency*.” Additional domains, such as familiarity and comfort, were indeed also mentioned in our study, but as subdimensions. These domains might have greater relevance for perceived safety, considering that the domains that we added, were probably related to a different set of DTs and the inclusion of both the HCP and HCR perspectives.

Our results showed that CDIFs were often related to each other from both the ES and PS perspectives. The finding that user friendliness was the most common factor in the domain “design and handling” was in line with the findings of Cimperman et al. [35]. User friendliness supports older adults’ acceptance and adoption of telecare.

The studies by Nyholm et al. [12]. and Akalin et al. [10] indicated that robots must be reliable and predictable in terms of their actions for patients to experience a sense of safety. DT reliability was also mentioned by Johannessen [11] in relation to the perceived safety of telecare and homecare professionals. In our study, we observed that reliable functionality is related to all other DTs that we have considered and that it should fit with the user’s abilities, skills, and resources. However, in our study, the predictability was also rather specific to robotics, from an HCP perspective.

Control was also mentioned by Nyholm et al. [12]. Patients felt safe when they had control, e.g., over patient data. In addition, we were able to subdivide control into three sub aspects: “gaining control through the DT, being in control of the DT, and (gaining) control of the DT by analog backup/redundant measure.” This highlights the complexity of this influencing factor. Control of one’s own health through the use of DTs was often mentioned as a positive factor, but support from HCPs or family members was also always an influencing factor and need. As described by Zhou et al. [36], support plays a crucial role for elderly people. However, healthcare services lack the capacity for sustained assistance, making family members essential in helping elderly people adjust to the digital society [36]. We saw a need for support for several target groups and that resources were seen as a clear perquisite for PS. For HCPs, not only the PS and its associated influencing factors but also the ES of the HCRs were important. Of the seven HCPs, six also considered ES in the context of PS. Considering our findings, perceived safety during DT use deserves increased attention given the ES, PS and implementation consequences of interactions with DTs. Perceived safety in relation to DTs influences the usage behavior, thoughts, emotions, and needs of HCPs and HCRs, with low perceived safety leading to cautious or altered use and strong perceived safety encouraging regular use.

### Limitations

Our research might have guided the focus of the participants only to perceived safety. However, our methods (e.g., think-aloud) provided the opportunity for other aspects related to the use of DTs. We were not able to cover all DTs according to the WHO classifications, but we had included a sample considering the perspectives of HCRs and HCPs and selected a variety of DTs in different settings. Credibility (validity) was increased by member checking during data collection, confirming the participants’ responses and statements regarding their perceived safety. The results were triangulated later in the overall study to enhance dependability (reliability).

### Conclusion

Perceived safety can have various consequences for further actions, feelings, and thoughts related to DT use. By considering the thirteen domains, we were able to identify core factors and needs in the context of perceived safety and DTs. When dealing with several DTs, special attention should be given to the context of perceived safety, target groups, ES and PS perspectives, settings, and DTs. ES and PS were determining factors in the acceptance of DT use and, therefore, the implementation success of DTs. To facilitate the adoption of these DTs, addressing these emotional needs becomes particularly important. The unmet needs of vulnerable HCRs should be considered because they often feel overwhelmed, uncertain, or insecure when faced with DTs, especially if they lack support. It is imperative to acknowledge these concerns to enhance ES. However, the interrelation of the PS with ES should also be considered. Finally, involving people from the early stages of developing DTs can help identify ES and PS needs and usability requirements and should be integral to the decision-making process of DT design in healthcare. Further research investigating quantitatively the relationship between the outcomes of increased or decreased perceived safety is needed.
