# Supplementary material for: Emotional and Psychological Safety in Healthcare Digitalization: A Design Ethnographic Study
Source: Int J Public Health. 2024 Sep 6;69:1607575. doi: 10.3389/ijph.2024.1607575 (PMC11414544; doi:10.3389/ijph.2024.1607575)
Supplement: Supplementary file 1 [file DataSheet2.docx]

| Supplemental file 2a: Outcomes in the context of emotional and psychological safety and digital technologies (North Rhine-Westphalia, Germany. 2023) | | | | | | | | | | | | | | |
| --- | --- | --- | --- | --- | --- | --- | --- | --- | --- | --- | --- | --- | --- | --- |
| **Outcomes** | **Main categories**  ***(Associated influencing factors)*** | **Subcategories**  ***(Use case examples)*** | **Digital technology** | | | | | | | | | | **TA** | **TG** |
|  |  |  | **1** | **2** | **3** | **4** | **5** | **6** | **7** | **8** | **9** | **10** |  |  |
| ***Outcomes of a low perceived safety in the context of digital technologies*** | | | | | | | | | | | | | | |
| Consequences for the implementation of DT | Digital technology is not used (limited competence, discomfort, risk perception, limited control) | Lack of technical affinity/knowledge leads to nonuse of the app. | x |  |  |  |  |  |  |  |  |  | + | A |
|  |  | Constantly visible heart rate values lead to discomfort and nonuse of Smart Watch/Apple Watch. |  |  | x |  |  |  |  |  |  |  | + | A |
|  |  | Surgery for chip implant for ECG measurement is seen as a risk and psychological burden and leads to nonuse of this option. |  |  | x |  |  |  |  |  |  |  | + | A |
|  |  | Individual and spontaneous reaction of the HCP (activation), only possible to a limited extent in the digital setting, leads to nonuse of DT (only used in Corona times/special exceptions). |  |  |  |  |  |  |  |  | x |  | +/- | HCP |
|  | Digital technology is used other than intended (should vs. is application) (limited functionality)° | Connection and transmission problems when changing the sensor, patient deletes the sensor data on the pump to avoid problems. |  |  |  | x |  |  |  |  |  |  | + | C |
|  | Digital technology is used, depending on certain circumstances (safety concerns, fear, pain)° | Concerns about data security and privacy lead to the app only being used in own secure WLAN. | x |  |  |  |  |  |  |  |  |  | + | A |
|  |  | Sensor change triggers pain and leads to the additional application of an ointment to reduce pain. |  |  |  | x |  |  |  |  |  |  | + | FM |
|  |  | Fear of using the VR glasses/robot alone/independently (e.g. fear of breaking something/doing something wrong) leads to conditional use with another person. |  |  |  |  | x | x |  |  |  |  | - | E |
|  |  | Concerns about the potential distraction of other programs on the laptop lead to the consequence of closing all programs before starting therapy. |  |  |  |  |  |  |  | x |  |  | + | A |
|  |  | Weighing up the benefits of a digital therapy session depending on the patient's age and disorder leads to the use or non-use of DT. |  |  |  |  |  |  |  |  | x |  | +/- | HCP |
|  | Digital technology is partially used (limited functionality, danger, limited control, limited competence and knowledge)° | Independent/autonomous navigating system of the robot is not fully developed (danger of driving over a person/falling down stairs) leads to non-use of this function. |  |  |  |  |  | x |  |  |  |  | + | HCP |
|  |  | Lack of control of the doctor's admission note via the dictation function of DT leads to non-use of this function (preferred use: analog writing on paper and independent typing on the computer). |  |  |  |  |  |  | x |  |  |  | - | HCP |
|  |  | Little experience with partial functions of the equipment leads to uncertainty and non-use of these functions. |  |  |  |  |  |  |  |  |  | x | + | HCP |
| Outcomes for HCR | The feeling of reduced control over DT | Forwarding to external sites has an impact on the patient's well-being e.g. having no control, not being able to decide freely. | x |  |  |  |  |  |  |  |  |  | + | A |
|  | Skepticism towards DT | Uncertainty about data security and forwarding leads to skepticism about the app. | x | x |  |  |  |  |  |  |  |  | + | A |
|  |  | Redirection to external sites leads to concerns/skepticism about DT. | x |  |  |  |  |  |  |  |  |  | + | A |
|  | Concerns related to DT° | Independent use of the pump leads to anxiety and insecurity. |  |  |  | x |  |  |  |  |  |  | + | C |
|  | Discomfort with DT | Functions of robotic such as playing music and following trigger a feeling of eeriness. |  |  |  |  |  | x |  |  |  |  | - | E |
|  | Feeling alienated by DT° | Not knowing how to use the robot triggers uncertainty and leads to a feeling of alienation. |  |  |  |  |  | x |  |  |  |  | - | E |
| Outcomes for HCP | Skepticism towards DT | Uncertainty about the benefits/effect of the app leads to skepticism. |  | x |  |  |  |  |  |  |  |  | + | HCP |
|  | Mistrust of DT | Missing warnings in the system trigger uncertainty which leads to mistrust of the system. |  |  |  |  |  |  | x |  |  |  | - | HCP |
|  | Emotional stress due to uncertainty | Lack of and limited competence in dealing with DT leads to a feeling of being overwhelmed in dealing with DT. |  |  |  |  |  |  |  |  |  | x | + | HCP |
| ***Outcomes of an increased perceived safety in the context of digital technologies*** | | | | | | | | | | | | | | |
| Consequences for the implementation of DT | Digital technology is used (recommendations, trust, positive (health) effects, easy handling) | Health insurance company recommendation as a quality indicator convinces critical patient to use the APP. | x |  |  |  |  |  |  |  |  |  | + | A |
|  |  | Evaluation and evidence of the app in the German Medical Journal and certification leads to the app being prescribed. |  | x |  |  |  |  |  |  |  |  | + | HCP |
|  |  | Positive effects of the app in terms of health impact led to the app being prescribed again. |  | x |  |  |  |  |  |  |  |  | + | HCP |
|  |  | Positive effects of the app lead to continuous use and extension of the app. |  | x |  |  |  |  |  |  |  |  | +/- | A |
|  |  | Familiar relationship with the therapist increases trust in DT. |  |  |  |  |  |  |  | x |  |  | + | A |
|  |  | Uncomplicated and easy handling of the digital video consultation. |  |  |  |  |  |  |  |  | x |  | +/- | HCP |
| Digital technology (DT): 1 = diet app, 2 = sleep app; 3 = mobile electrocardiogram; 4 = closed-loop-system pump; 5 = virtual reality; 6 = robotic; 7 = hospital information system; 8 = telemedicine psychotherapy; 9 = tele-psychotherapy; 10 = simulation training in emergency care, Technical affinity (TA): + = higher technical affinity; - = lower technical affinity; Perspective of target groups (TG): HCR = healthcare recipients (A = adult, E = elderly, C = child), FM = family member, HCP = healthcare provider | | | | | | | | | | | | | | |

| Supplemental file 2b: Influencing factors in the context of emotional safety and digital technologies (visit 1,2,3 including feedback loop) | | | | | | | | | | | | | | | | | | | |  |
| --- | --- | --- | --- | --- | --- | --- | --- | --- | --- | --- | --- | --- | --- | --- | --- | --- | --- | --- | --- | --- |
| **Core dimension** | **Influencing factors in the context of emotional safety and digital technology** |  | **Digital technology** | | | | | | | | | | | | |  | | | |  |
|  | **Main categories** | **IF** | | **1** | **2** | **3** | **4** | **5** | | **6** | | **7** | **8** | **9** | **10** | | **TA** | | **TG** | |
| **Domain: Support** | | | | | | | | | | | | | | | | | | | | |
| Support in the context of DT | Support during the use of the DT by HCP or DT. | + | | x | x |  |  | |  | |  |  |  |  |  | | | +,+/- | A | |
|  | Uncertainty in independent use of the DT (without support) (uncertainty regarding correct use and ending of the DT). | - | |  |  |  | x | | x | |  |  |  |  |  | | | -, + | E, FM | |
|  | Lack of contact person on personal health status via DT. | - | | x |  |  |  | |  | |  |  | x |  |  | | | + | A | |
| Effective inclusion of medical expertise by DT | DT enables (flexible/real-time) inclusion of medical expertise (data transmission, feedback from HCP, communication between HCR and HCP about health data in DT). | + | | x |  | x |  | |  | |  |  |  |  |  | | | + | A | |
| **Domain: Autonomy of RECIPIENTS** | | | | | | | | | | | | | | | | | | | | |
| Autonomy and participation by DT | Self-management by DT (DT creates sovereignty e.g. by experience). | + | |  |  | x |  | |  | |  |  |  |  |  | | | + | A | |
|  | DT allows room for possible actions and own initiative (self-organization). | + | | x |  | x |  | |  | |  |  | x |  |  | | | + | A | |
| Digital technology (DT): 1 = diet app, 2 = sleep app; 3 = mobile electrocardiogram; 4 = closed-loop-system pump; 5 = virtual reality; 6 = robotic; 7 = hospital information system; 8 = telemedicine psychotherapy; 9 = tele-psychotherapy; 10 = simulation training in emergency care, Technical affinity (TA): + = higher technical affinity; - = lower technical affinity; Perspective of target groups (TG): HCR = healthcare recipients (A = adult, E = elderly, C = child), FM = family member, HCP = healthcare provider * = Reference to emotional or psychological safety explicitly mentioned; influencing factors (IF): + = facilitating factors, - = inhibitory factors, 0 = neutral | | | | | | | | | | | | | | | | | | | | |

| Supplemental file 2c: Influencing factors in the context of psychological and emotional safety and digital technologies (visit 1,2,3 including feedback loop) (North Rhine-Westphalia, Germany. 2023) | | | | | | | | | | | | | | |
| --- | --- | --- | --- | --- | --- | --- | --- | --- | --- | --- | --- | --- | --- | --- |
| **Core dimension** | **Influencing factors in the context of psychological and emotional safety and digital technologies** | | **Digital technology** | | | | | | | | | | **TA** | **TG** |
|  | **Main categories** | **IF** | **1** | **2** | **3** | **4** | **5** | **6** | **7** | **8** | **9** | **10** |  |  |
| **Domain: Design & handling** | | | | | | | | | | | | | | |
| Health and need orientated use of DT | Target group-specific adaptability of DT to the HCR's needs and expectations (e.g. adaptability to the patient's affinity for technology, cognitive and motor skills). | + |  |  |  |  | x |  |  |  |  |  | + | HCP |
|  | Interest in the DT depending on the HCR's character traits, e.g. curiosity. | 0 |  |  |  |  | x |  |  |  |  |  | - | E |
|  | Risks and stress by DT. | 0 |  |  | x |  |  |  |  |  |  |  | + | A |
|  | Habit-oriented digital usage arrangement. | + |  |  |  |  | x |  |  | x |  |  | + | HCP, A |
|  | Dropping of the usual rituals in the design of the framework conditions of the health service by DT. | - |  |  |  |  |  |  |  | x |  |  | + | A |
|  | Limitation of DT use due to illness. | - |  | x |  |  | x |  |  |  |  |  | +/-  - | A, E |
|  | Need for individual consideration of the suitability of the DT regarding the HCR. | 0 |  |  |  |  | x |  |  |  | x |  | +  +/- | HCP |
|  | DT ensures that safety measures (psychological/physical) are individually adapted to the HCR's state of health. | + |  | x |  |  |  |  |  |  |  |  | + | HCP |
|  | Need-oriented and flexible availability and usability of DT. | + |  | x |  |  |  |  |  |  |  |  | +/- | A |
|  | Need-based transmission of measured health data by DT. | + |  |  | x |  |  |  |  |  |  |  | + | A |
| User-friendliness of DT | Easy handling of the DT, e.g. through illustration/visualization (own initiative). | + | x | x | x | x |  |  |  | x | x |  | +  +/- | A, HCP, FM, C, |
|  | Clarity of the DT, e.g. patient data°. | + |  |  |  |  |  |  | x |  |  |  | - | HCP |
|  | Concerns about the practicability of the DT (user-friendliness of the DT). | - |  |  |  |  |  |  | x | x |  | x | -, + | HCP, A |
| Flexible availability and useability of DT | Flexible availability and usability of the DT by hardware and software design (possibility to carry and use DT flexibly - increases HCR motivation). | + | x | x | x |  |  |  |  | x |  |  | +/-, + | A, HCP |
|  | Suitability of the DT for everyday use. | + |  |  | x |  |  |  |  |  |  |  | + | A |
|  | Limitations and challenges of information perception and recording in different situations (at night, during sport). | - |  |  |  | x |  |  |  |  |  |  | + | C, FM |
|  | DT (robotics) enables individual and flexible care options. | + |  |  |  |  |  | x |  |  |  |  | + | HCP |
|  | DT (Virtual Reality) enables planning security through individual and flexible care options. | + |  |  |  |  | x |  |  |  |  |  | + | HCP |
| Customizability of DT | Saving HCR's experiences of use and competences in dealing with the DT in DT.  (for the coordination of an individually suitable use)°. | + |  |  |  |  | x |  |  |  |  |  | + | HCP |
|  | Customizable design/functions of the DT (for identification/incentive). | + | x | x |  |  |  |  |  |  |  |  | +/-, | A |
|  | Lack of customizability of the DT, e.g. individualized information in the DT is not possible (such as food intolerances)* | - | x |  |  |  |  |  |  |  |  |  | + | A |
| Setting of DT | Disturbances caused by the digital setting/by the environment. | - |  |  |  |  |  |  |  | x | x |  | +  +/- | A, HCP |
|  | Disturbance-free setting when using the DT independent of external conditions, e.g. distraction by residents or telephone ringing°* | + |  |  |  |  | x |  |  |  |  |  | -  + | E,  HCP |
|  | Design freedom of the setting in relation to DT. | + |  |  |  |  |  |  |  | x |  |  | + | A |
|  | Disruptions caused by the DT in everyday life of HCR. | - |  |  |  |  |  |  |  | x |  |  | + | A |
| Standardization of DT | Non-standardized measured variables of the DT (e.g. serving size in diet app). | - | x |  |  |  |  |  |  |  |  |  | + | A |
| technical integrity of the DT | Confidence in the integrity of the technology during use e.g. VR headset. | + |  |  |  |  | x |  |  |  |  |  | - | A |
|  | Expectation to damage the DT during usage* | - |  |  |  |  |  | x |  |  |  |  | -  + | A, HCP |
| Perceptible high quality of DT | Professional design and display of the DT. | + |  |  |  |  |  |  |  | x |  |  | + | A |
|  | DT classified and perceived as high quality. | + |  | x |  |  |  |  |  |  |  |  | + | HCP |
|  | Content design and organization of the DT. | + |  | x |  |  |  |  |  |  |  |  | +/- | A |
| **Domain: Knowledge & competence** | | | | | | | | | | | | | | |
| Self-confidence when using DT | Overwhelmed by DT, e.g. due to the complexity of the DT and a lack of knowledge about DT. | - |  |  |  |  |  |  |  |  |  | x | + | HCP |
|  | (Lack of) self-confidence in one's own competence in dealing with the DT.°* | +/- |  |  |  | x | x |  |  |  |  |  | -,+ | E, C |
| Providers' competence and knowledge towards the DT | Existing competences in relation to the DT. * | + |  |  |  |  |  |  |  |  |  | x | + | HCP |
|  | Lack of competence and knowledge in dealing with the DT. | 0 |  |  |  |  |  |  |  |  |  | x | + | HCP |
|  | Professional experience of the HCP. | 0 |  |  |  |  |  |  |  |  |  | x | + | HCP |
| Recipients' knowledge and competence towards DT | Existing competences in relation to the DT* | + |  |  |  |  |  | x |  |  |  |  | -  + | E, HCP |
|  | Technical socialization of the HCR facilitates easier handling of the DT.°* | + |  |  |  |  | x |  |  |  |  |  | + | HCP |
|  | DT enables and requires a reflection on personal health status and behavior, e.g. digital diary for self-reflection. | 0 | x |  |  |  |  |  |  |  |  |  | + | HA |
|  | Lack of competence and knowledge in dealing with the DT. | - |  |  |  |  | x | x |  |  |  |  | - | E |
|  | Concerns about HCR's ability to handle/operate the DT, e.g. insufficient motor skills, stress or dementia. | - |  |  |  |  | x | x |  |  |  |  | -, + | E, HCP |
|  | Need-based knowledge and competence acquisition of HCR and FM by DT. | + |  | x |  |  |  |  |  |  |  |  | +, +/- | A, HCP |
|  | Knowledge about possibilities and limits of DT. | + |  |  |  |  | x |  |  |  |  |  | + | HCP |
|  | Too much/overwhelming amount of information. | - |  | x |  |  |  |  |  |  |  |  | +/- | A |
|  | Lack of clarity on data processing, interpretation, security, technology, treatment procedures with DT, ethics, government information policy. | - | x |  |  | x |  |  |  | x |  |  | + | A, C, |
| Familiarity by regularity of use | Regular use of the DT to increase the competence, familiarity & handling/usage confidence of the users. | + |  | x | x |  | x | x |  | x | x | x | -, +, +/- | E, HCP, A |
|  | Irregularity of the DT usage arrangement (HCR willingness to participate, HCR state of health). | - |  |  |  |  | x |  |  |  |  |  | + | HCP |
| **Domain: Technical functionality** | | | | | | | | | | | | | | |
| (Un)reliable functionality of the DT | Reliable functionality of the DT. | + |  | x |  | x | x | x |  | x | x |  | -, +  +/- | E, A, HCP, C |
|  | Interference with DT by HCR (interference is expected, lack of skills, limited senses). | - |  |  | x | x |  |  |  | x |  |  | + | A, HCP |
|  | Susceptibility of the DT to malfunction (unreliable functionality). | - | x |  | x |  | x |  | x | x |  | x | -, +  +/- | HCP, A |
|  | Delayed or unreliable diagnosis by DT. | - |  |  | x |  |  |  |  |  |  |  | + | A |
|  | Correctly recorded health data of HCR by DT. | + |  |  |  |  |  | x |  |  |  |  | + | HCP |
|  | Expectation of misdiagnosis/treatment due to unreliable or missing information linkage and transmission of the DT. (missing warning, missing processing status). | - |  |  |  |  |  |  | x |  |  |  | - | HCP |
|  | Reliability of digital health data and its transmission to HCP (to ensure correct/appropriate diagnosis & treatment). | + |  | x |  |  |  |  | x |  |  |  | -, + | HCP |
|  | Ensuring the availability of patient data via various digital accesses. | + |  |  |  |  |  |  | x |  |  |  | - | HCP |
|  | Planning security by technical reliability of the DT, e.g. error-free functionality, feedback function of the system of the DT.* | + |  |  |  |  |  | x |  |  |  |  | + | HCP |
|  | Predictability of DT. | - |  |  |  |  |  | x |  |  |  |  | + | HCP |
| Autonomous function of DT in healthcare | Autonomous function of the DT to avoid human error. | + |  |  |  | x |  |  | x |  |  |  | +,- | FM |
|  | Technically autonomous (warning) functions, diagnostics, treatment, and monitoring of health status by DT. | + |  | x |  | x |  |  |  |  |  |  | +, +/- | FM, C, A |
| **Domain: Recognition of social interaction** | | | | | | | | | | | | | | |
| Recognition of interpersonal interaction | Need for interpersonal interaction with patients to promote the doctor-patient relationship e.g. through eye contact* | + |  |  |  |  |  |  | x |  |  |  | - | HCP |
|  | Exchange about the experience of the DT between HCR and HCP. | + |  |  |  |  |  |  |  |  | x |  | +/- | HCP |
|  | Comparison of expectations towards DT between HCP and HCR via feedback loop°* | + | x | x |  |  |  |  |  |  |  |  | + | HCP, A |
|  | Reduction or loss of familiar interpersonal interactions/relationships when using the DT. | - |  |  |  |  |  | x |  | x | x | x | + | A, HCP |
|  | Perception of inadequate health service by DT due to limited interpersonal interaction. | - |  |  |  |  |  |  |  | x | x |  | +  +/- | A, HCP |
|  | Possibility of familiar visual interaction in parts possible by DT. | + |  |  |  |  |  |  |  | x | x |  | +  +/- | A, HCP |
| **Domain: Data protection, transparency & security** | | | | | | | | | | | | | | |
| Secure data management & protection | Data protection indicator, certification and ensuring data protection of DT and by DT. | + |  | x |  |  |  |  |  |  | x |  | +, +/- | HCP |
|  | Data security with individual authentication, e.g. when accessing the DT.* | + | x |  |  |  |  |  |  |  |  |  | + | A |
|  | Data protection and data security for users of the DT through automatic data deletion by the DT, e.g. to prevent the inspection and misinterpretation of data by FM. | + |  |  |  |  |  | x |  |  |  |  | + | HCP |
|  | Data protection regulations not respected in the handling of health data. | - | x |  |  |  |  |  |  |  |  |  | + | A |
|  | Data protection and data security concerns of DT users. | - | x |  |  |  |  | x |  | x |  |  | + | HCP, A |
|  | Weighing up the use of the DT with regard to data protection concerns in relation to the benefit of the DT (usefulness of the DT despite the restriction of data protection)* | - | x |  | x |  |  |  |  |  |  |  | +  + | A |
|  | need to disclose sensitive data in DT (compared to analogue therapy/treatment). | 0 |  | x |  |  |  |  |  |  |  |  | +/-  + | A,  HCP |
|  | Lack of clarity about the backup and protection of personal patient data in the DT. | - | x | x |  |  |  |  |  |  |  |  | +, +/- | A |
| Anonymity & privacy in DT use | Expected restriction of data protection, anonymity and privacy when using the DT. | - | x |  |  |  |  |  |  |  |  |  | + | A |
|  | Open minded towards the function and design of the DT through modern technology e.g. camera and microphone. | 0 |  |  |  |  |  | x |  |  |  |  | - | E |
|  | Necessity of data transfer as a requirement for an extended function of the DT. | 0 |  |  |  |  |  |  |  | x |  |  | + | A |
|  | Weighing the risk of poor data security vs. the sensitivity of the potentially accessible health data* | 0 | x |  |  |  |  |  |  |  |  |  | + | A |
|  | Restriction of HCR privacy by DT, e.g. possibility of crossing HCR privacy boundary when extending digital camera field of view° | - |  |  |  |  |  |  |  |  | x |  | +/- | HCP |
|  | Protect the HCR's privacy (respect the HCR's boundaries), e.g. when extending the digital camera field of view in the child's room during online therapy session, e.g. discretion when using the DT in everyday life. | 0, + | x |  |  |  |  |  |  |  | x |  | +/-  + | HCP, A |
|  | Selection of the DT in relation to knowledge about and consideration of data security. | 0 |  | x |  |  |  |  |  |  |  |  | + | HCP |
| Transparency (of data management) of DT | Lack of transparent management of digital data by DT. | - |  | x |  |  |  |  |  |  |  |  | + | HCP |
|  | Transparency of DT (own health data & data processing). | + | x | x |  |  |  |  |  |  |  |  | +/-, + | A |
|  | Transparent data management with the possibility of independent data editing of the HCR (data sovereignty), e.g. by cloud with option to edit in real time.°. | + |  | x |  |  |  |  |  |  |  |  | + | HCP |
|  | Insufficient transparency of the processing status within the DT. | - |  | x |  |  |  |  | x |  |  |  | +, - | HCP |
| **Domain: Control** | | | | | | | | | | | | | | |
| Gaining control through the DT | Monitoring of own health status by professionals and/or caregivers with the help of the DT  (e.g. by monitoring the state of health). | + | x |  |  | x |  |  |  |  |  |  | + | C, FM, A |
|  | Health monitoring by DT (feeling of being protected/independent from the disease). | + |  |  |  | x |  |  |  |  |  |  | + | C, FM |
|  | DT enables verifiability of health services. | + |  | x |  |  |  |  |  |  |  |  | +/- | A |
|  | Control of own digital health data by DT (personal health status and health behavior). | + | x |  | x |  |  |  |  |  |  |  | + | A |
| Being in control of the DT | Control over one's own health data in the DT (data sovereignty). | + |  |  |  |  |  | x |  |  | x |  | +  +/- | HCP |
|  | Lack of control over personal health data in the DT (necessary acceptance of limited data sovereignty for the benefit of treatment). | - | x |  |  |  |  |  |  | x |  |  | + | A |
|  | Control and influenceability of the DT during usage by HCP and HCR. | + |  |  |  |  | x |  |  |  |  | x | -,+ | E, HCP |
|  | Weighing of greater trust in the HCP vs. less ability to influence the DT. | 0 |  |  |  |  |  |  |  | x |  |  | + | A |
|  | Possibilities for patient to manipulate DT. | - |  | x |  |  |  |  |  |  |  |  | + | HCP |
|  | Controllability of the functionality of the DT through information displays of the DT. | + |  |  |  | x |  |  |  |  |  |  | + | C |
|  | Insufficient controllability of the DT. | - |  | x |  |  |  |  | x |  |  |  | +, - | HCP |
|  | Lack of control over HCR to ensure integrity of HCR and DT.°* | - |  |  |  |  | x |  |  |  |  |  | + | HCP |
|  | Escalation, during use of the DT poses threat e.g. triggered by nervousness (not further defined). | - |  |  |  |  |  |  |  |  |  | x | + | HCP |
|  | Power of decision by HCP towards DT.° | + |  |  |  |  |  |  | x |  |  |  | - | HCP |
| (Gaining) control over the DT through analogue back-up/redundant measures | Additional analogue/manual measures for safeguarding in addition to the DT (continuous). | + |  |  |  | x |  |  |  |  |  |  | + | C |
| **Domain: Resources** | | | | | | | | | | | | | | |
| Resource availability & management regarding DT | Resource-efficient care services by DT. | + |  |  |  |  | x |  |  |  |  |  | + | HCP |
|  | Limited time resources to (regularly) discuss the use of the DT, e.g. appointment capacities of HCP and HCR° | - |  | x |  |  |  |  |  |  |  |  | +/-  + | A, HCP |
|  | Economic resource scarcity of the HCP. | - |  |  |  |  | x |  |  |  | x |  | + | HCP |
|  | HCP resource scarcity due to time and staffing limitations. | - |  | x |  |  | x |  |  |  | x | x | +, +/- | HCP |
| **Domain: Efficiency & effects** | | | | | | | | | | | | | | |
| Efficient healthcare provided by DT | Relief of healthcare system by DT: Efficiency of health services by DT. | + |  |  | x |  |  |  | x |  |  | x | -, + | HCP, A |
|  | Efficient and rapid transmission of health data (to HCP) by DT (for reporting and diagnosis by HCP). | + |  |  | x |  |  |  | x |  |  |  | +, - | A, HCP |
|  | Enable continuous use of health services by DT, taking into account the pandemic situation/health status. | + |  | x |  |  |  |  |  | x | x |  | +/-  + | A, HCP |
|  | Enabling the continuous use of health services by DT in cases of urgent need. | + |  |  |  |  |  |  |  | x |  |  | + | A |
|  | Gaining additional information about HCR through DT, e.g. extended therapeutic insight into the HCR's living environment through digital setting* | + |  |  |  |  |  |  |  |  | x |  | +/- | HCP |
|  | Constant exchange with colleagues on HCR health data and treatment status partly via DT.° | + |  |  |  |  |  |  | x |  |  |  | - | HCP |
|  | Challenges and concerns about the effort and involved time using DT. | - |  |  |  |  |  |  |  |  | x | x | +,  +/- | HCP |
|  | Price advantage of DT over analogue applications. | 0 |  |  |  |  |  |  |  |  |  | x | + | HCP |
|  | Close contact with the DT company, e.g. to improve processes in the use of DT. | 0 |  |  |  |  |  |  |  |  |  | x | + | HCP |
|  | Ensuring care in emergencies by DT (efficient and effective data transfer and access). | + |  |  | x |  |  |  |  |  |  |  | + | A |
|  | Efficient access to health data by DT. | + |  |  | x |  |  |  |  |  |  |  | + | A |
| Health and care effects associated with DT | Target group-oriented health-promoting tasks for patients/residents by functions of DT. | + |  | x |  |  |  |  |  |  |  |  | +/-  + | HCP, A |
|  | Expectation of a lack of suitability of the DT in healthcare. | - |  |  |  |  |  |  |  | x |  |  | + | A |
|  | Concerns about the physical/mental well-being of HCRs and HCPs during the use of the DT. | - |  | x |  |  | x |  |  |  |  |  | + | HCP |
|  | Suggestion of practical everyday exercises for symptom relief by DT°*, e.g. relaxation exercises. | + |  | x |  |  |  |  |  |  |  |  | +/- | A |
|  | Perception of higher chances of health success by DT. | + |  | x |  |  |  |  |  |  |  |  | +/- | A |
|  | Gained acceptance towards the HCR's disease through knowledge transfer of the DT.* | + |  | x |  |  |  |  |  |  |  |  | +/- | A |
| Accessibility by DT | DT creates independence regarding required barrier-free access for HCR in the realization of care offers of the HCP (VR). | + |  |  |  |  | x |  |  |  |  |  | + | HCP |
| **Domain: Trust** | | | | | | | | | | | | | | |
| Trust in and by providers in the context of DT | Use of the DT requires familiar/existing relationship between HCP and HCR. | + |  |  |  |  |  |  |  | x | x |  | +, +/- | A, HCP |
|  | Trust in DT's IT department regarding data encryption* | + |  |  |  |  |  |  |  |  | x |  | +/- | HCP |
|  | HCP as mediator for trust in DT (reference person, emphatic and credible interaction and transfer of knowledge builds trust).  (Trust in human beings is higher than in technology). | + | x |  |  | x | x | x |  | x |  |  | -, + | E, HCP, C, A |
| **Domain: Prerequisites** | | | | | | | | | | | | | | |
| Readiness of RECIPIENTs to implement and get used to DT | Consent of HCR before usage of DT. | + |  |  |  |  |  | x |  |  |  |  | - | E |
|  | Need to acquire the DT-technical usage as a prerequisite for DT use° | 0 |  |  |  |  | x |  |  |  |  |  | + | HCP |
| Usage& implementation concerns associated with DT | Implementation and realization concerns related to DT (legal, technical, financial, lack of digital therapy concepts). | - |  |  |  |  |  |  | x | x | x |  | +/-  +,- | HCP, A |
|  | Need to be proactive in adapting analogue methods for healthcare services when using DT. | 0 |  |  |  |  |  |  |  |  | x |  | +/- | HCP |
|  | Usage and implementation concerns about the DT (staff-related, data protection concerns in relation to the benefits of the DT). | - | x | x |  |  |  | x |  |  |  |  | -, + | E, HCP, A |
|  | Self-discipline and sincerity as a prerequisite for successful use of the DT. | 0 |  |  |  |  |  |  |  | x |  |  | + | A |
|  | Skepticism about the benefits of DT (compared to analogue medical treatment). | - |  | x |  |  |  |  |  |  |  |  | + | HCP |
|  | Interest and consideration of equivalence of analogue vs. digital treatment (therapy session). | 0 |  |  |  |  |  |  |  | x |  |  | + | A |
|  | Preparatory strategies for potential problems during the use of the DT. | + |  |  |  |  |  |  |  |  | x |  | +/- | HCP |
|  | Preparatory strategies and arrangements before using the DT. | + |  |  |  |  |  |  |  | x |  | x | + | HCP, A |
|  | Implementation perquisites e.g. Legal restrictions choosing DT. | - | x |  |  |  |  |  |  | x |  |  | + | A |
| Freedom from pain & physical safety when using DT | Freedom of pain while use of the DT, e.g. local anesthetics during sensor/catheter change.° | + |  |  |  | x |  |  |  |  |  |  | + | C |
|  | Certainty about the physical well-being of the HCR and HCP during the use of the DT (hazard exclusion).°* | + |  |  |  |  | x |  |  |  |  | x | + | HCP |
| **Domain: Experience & perspective** | | | | | | | | | | | | | | |
| Experiences | (Positive) previous experience with a known or similar DT. | + |  |  |  |  |  | x |  | x |  |  | -, + | E, HCP, A |
|  | (Negative) previous experience with DT without health reference. | - |  |  |  |  |  |  |  | x |  |  | + | A |
|  | Absent/Low/Negative experience with the DT.°* | - |  | x |  |  |  |  |  |  |  | x | + | HCP |
| Attitudes towards DT | HCP's affinity for technology in relation to DT. | + |  |  |  |  |  | x |  |  |  |  | + | HCP |
|  | Lack of interest in the DT. | - |  |  |  |  |  | x |  |  |  |  | - | E |
| (Negative) feelings during the use of DT | Feeling of eeriness or discomfort in relation to functions of the DT e.g. following function and playing music° or realistic, visual high-altitude representation of the VR headset. | - |  |  |  |  | x | x |  |  |  |  | - | E |
| Reputational concerns | Concerns about own or organizational reputation due to data transmission errors or errors caused by the DT. | - |  |  |  |  |  |  | x |  |  |  | - | HCP |
| Digital technology (DT): 1 = diet app, 2 = sleep app; 3 = mobile electrocardiogram; 4 = closed-loop-system pump; 5 = virtual reality; 6 = robotic; 7 = hospital information system; 8 = telemedicine psychotherapy; 9 = tele-psychotherapy; 10 = simulation training in emergency care, Technical affinity (TA): + = higher technical affinity; - = lower technical affinity; Perspective of target groups (TG): HCR = healthcare recipients (A = adult, E = elderly, C = child), FM = family member, HCP = healthcare provider * = Reference to emotional or psychological safety explicitly mentioned; influencing factors (IF): + = facilitating factors, - = inhibitory factors, 0 = neutral | | | | | | | | | | | | | | |

| Supplemental file 2d: Needs in the context of emotional and psychological safety and digital technology (North Rhine-Westphalia, Germany. 2023) | | | | | | | | | | | | | |
| --- | --- | --- | --- | --- | --- | --- | --- | --- | --- | --- | --- | --- | --- |
| **Core dimension of needs (CDN)** | **Main categories** | **Digital technology** | | | | | | | | | | **TA** | **TG** |
|  |  | **1** | **2** | **3** | **4** | **5** | **6** | **7** | **8** | **9** | **10** |  |  |
| **Needs in the context of emotional safety and digital technology** | | | | | | | | | | | | | |
| **Domain: Prerequisites** | | | | | | | | | | | | | |
| Equity of access to DT | Equity of access to DT (affordability, low-threshold, intergenerational equity & alternative access). |  |  |  | x |  |  |  |  |  |  | +  + | FM,  C |
| **Domain: Technical functionality** | | | | | | | | | | | | | |
| Autonomous function of DT in healthcare | Autonomous function of diagnosis & treatment of DT. |  |  |  | x |  |  |  |  |  |  | + | FM, C |
| **Domain: Knowledge & competence** | | | | | | | | | | | | | |
| Self-confidence when using DT | Self-confidence in one's own competence in dealing with the DT. |  |  |  |  | x |  |  |  |  |  | - | E |
| **Needs in context of psychological safety and digital technology** | | | | | | | | | | | | | |
| **Domain: Resources** | | | | | | | | | | | | | |
| Resource availability & management regarding DT | Use of the robot to promote a future-oriented management culture°* e.g. support for interactive care, improve and simplify planning and processes within the retirement home. |  |  |  |  |  | x |  |  |  |  | + | HCP |
| **Domain: Prerequisites** | | | | | | | | | | | | | |
| Usage & implementation concerns associated with DT | Building trust of HCR in DT for successful implementation° |  | x |  |  |  |  |  |  |  |  | + | HCP |
| **Domain: Design & handling** | | | | | | | | | | | | | |
| Technical integrity of the DT | Certainty that the DT is not damaged or will not be damaged by use°* |  |  |  |  | x |  |  |  |  |  | + | HCP |
| **Domain: Experience & perspective** | | | | | | | | | | | | | |
| Attitudes towards DT | Preference for analogue healthcare e.g. therapy sessions. |  |  |  |  |  |  |  |  | x |  | +/- | HCP |
| Irreplaceability of human in healthcare | Affirm the professional self-image of nurses and the necessity of the nursing profession (DT should not replace people/ HCP). |  |  |  |  |  | x |  |  |  |  | + | HCP |
| **Needs in context of emotional and psychological safety and digital technology** | | | | | | | | | | | | | |
| **Domain: Knowledge & competence** | | | | | | | | | | | | | |
| Recipients' knowledge and competence towards DT | Education and empowerment of HCRs/ FMs. |  |  |  | x | x |  |  |  |  |  | + | C, HCP |
| Providers' competence and knowledge towards the DT | Education and empowerment of HCP. |  |  |  |  | x |  |  |  |  |  | + | HCP |
| Familiarity by regularity of use | Habit through the regular use of and early introduction to the use of the DT. |  |  |  |  | x | x |  |  |  |  | +,- | HCP, E |
| **Domain: Efficiency & effects** | | | | | | | | | | | | | |
| Efficient healthcare provided by DT | Provision and transfer of information on the HCR's health data/feedback to the HCP. |  |  |  |  |  |  | x |  |  |  | - | HCP |
|  | Relief of the HCP by DT. |  |  |  |  |  | x | x |  |  | x | +,- | HCP |
|  | Ensuring healthcare with correct/proper diagnosis/treatment. |  |  |  |  |  |  | x |  |  |  | - | HCP |
| **Domain: Technical functionality** | | | | | | | | | | | | | |
| (Un)reliable functionality of the DT | Error-free and reliable functionality of the DT. |  |  |  | x | x | x | x |  | x | x | -,+  +/- | C, FM, E, HCP |
|  | Technical and infrastructural stability for a reliable use of DT, e.g. developed and functioning digital infrastructure. |  |  |  |  |  |  |  |  | x |  | +/- | HCP |
|  | Continuous, seamless recording of health-relevant data by the DT. |  |  |  | x |  |  |  |  |  |  | + | C, FM |
| **Domain: Design & handling** | | | | | | | | | | | | | |
| Setting of DT | Ensure a disturbance-free setting while using DT, e.g. through enough HCP staff, through FM, possibility of visual signaling for the need of undisturbed surroundings, have peace of mind through video-based simulation training. |  |  |  |  | x |  |  | x | x | x | +  +/- | HCP,  A |
|  | Spatial and equipment-specific specialization of the setting on the use of the DT, e.g. VR technology already installed, armchairs for residents. |  |  |  |  | x |  |  |  |  |  | + | HCP |
| Customizability of DT | Needs-oriented individualization of the DT. | x |  |  | x | x |  |  |  |  |  | + | HCP, FM, A |
|  | Digitization/applicability of analogue methods and materials in digital setting. |  |  |  |  |  |  |  |  | x |  | +/- | HCP |
|  | Individual design with regard to health, habits and interests of the users. |  | x | x |  | x |  |  | x |  |  | +, +/- | HCP, A |
| Optimization of DT | Technical optimization/expansion of the DT (functions, compatibility, alternatives, infrastructure). |  | x | x | x | x | x |  |  | x | x | +  +/- | A, HCP, C, FM |
| User-friendliness of DT | User-friendly design of the DT. |  |  | x | x | x |  |  |  |  |  | +, - | A; E, HCP |
|  | Easy handling of the DT, e.g. through intuitive operation of the operating system, easier sending of measured health data. |  |  | x |  | x |  |  |  |  | x | + | A, HCP |
| Flexible availability and useability of DT | Feasibility and planning security of leisure activities for HCR through DT e.g. independence of barrier-free access options° |  |  |  |  | x |  |  |  |  |  | + | HCP |
|  | Suitability of the DT for everyday use (integrating the DT into everyday life/routine). |  |  | x | x |  |  |  | x |  |  | + | A, C |
|  | Technical independence and flexible accessibility of the DT. |  |  | x |  |  |  |  |  |  |  | + | A |
|  | Location-independent availability of familiar health services with person of trust enabled by DT. |  |  |  |  |  |  |  | x |  |  | + | A |
| **Domain: Data protection, transparency & security** | | | | | | | | | | | | | |
| Transparency (of data management) of DT | Transparency of DT on several levels (communication, information). | x | x |  |  |  |  |  |  |  |  | + | A, HCP |
| Secure data management & protection | Ensuring data protection. | x |  |  |  |  |  |  | x |  |  | + | A |
|  | Control over own health data (data sovereignty, data protection, data evaluation and data transfer). | x |  |  |  |  |  |  | x |  |  | + | A |
| Anonymity & privacy in DT use | Protection of privacy. |  |  |  |  |  | x |  | x |  | x | + | HCP, A |
| **Domain: Support** | | | | | | | | | | | | | |
| Support in the context of DT | Possibility to make use of external support (services) for HCP on DT, e.g. for technical questions. |  |  |  |  | x |  |  |  |  |  | + | HCP |
|  | Introduction to DT by familiar and trusted person. | x |  |  |  |  | x |  | x |  |  | -,+ | E, A |
|  | Support and assistance during the use of the DT. | x |  |  |  | x | x |  |  |  |  | -,+ | E, A, HCP |
| Health and care effects associated with DT | Physical relief of the HCR, e.g. voice-controlled opening of the door so that the robot can drive in.° |  |  |  |  |  | x |  |  |  |  | - | E |
|  | sense of safety conveyed by presence of DT. |  |  |  |  |  | x |  |  |  |  | + | HCP |
| **Domain: Control** | | | | | | | | | | | | | |
| Gaining control through the DT | Possibility to control one’s own state of health by DT. | x | x |  | x |  | x |  |  |  |  | +/-+,- | HCP, A, C |
|  | Control of the HCP's healthcare performance by DT. |  |  |  |  |  |  | x |  |  |  | - | HCP |
| Being in control of the DT | Power of decision by HCP towards DT.° |  |  |  |  |  |  | x |  |  |  | - | HCP |
|  | control of technology, e.g. through an emergency stop switch. |  |  |  |  | x |  |  |  |  |  | + | HCP |
|  | Control/control possibility by person of trust with the help of the DT. |  |  |  | x |  | x |  |  |  |  | +,- | C, FM, E |
| **Domain: Prerequisites** | | | | | | | | | | | | | |
| Freedom from pain & physical safety when using DT | Cross-disciplinary access to health data in the healthcare setting by DT, e.g. in emergency situations. |  |  | x |  |  | x |  |  |  |  | +  + | A, HCP |
|  | Ensuring healthcare in emergency and exceptional situations in the event of failure/restriction of the DT. | x |  |  |  |  |  |  |  |  |  | + | A |
|  | Certainty about the wellbeing of healthcare provider and receiver during use of the DT. |  |  |  |  | x |  |  |  |  |  | + | HCP |
| **Domain: Recognition of social interaction** | | | | | | | | | | | | | |
| Recognition of psycho-social care by DT | Personal benefits of DT: reviving memories and making personal connections, e.g. to childhood. |  |  |  |  | x |  |  |  |  |  | - | E |
|  | Further development of DT to support the healthcare, e.g. to promote the HCR's social participation by video call function of DT. |  |  |  |  |  | x |  |  |  |  | + | HCP |
| Digital technology (DT): 1 = diet app, 2 = sleep app; 3 = mobile electrocardiogram; 4 = closed-loop-system pump; 5 = virtual reality; 6 = robotic; 7 = hospital information system; 8 = telemedicine psychotherapy; 9 = tele-psychotherapy; 10 = simulation training in emergency care, Technical affinity (TA): + = higher technical affinity; - = lower technical affinity; Perspective of target groups (TG): HCR = healthcare recipients (A = adult, E = elderly, C = child), FM = family member, HCP = healthcare provider * = Reference to emotional or psychological safety explicitly mentioned | | | | | | | | | | | | | |
