# Supplementary material for: Emotional and Psychological Safety in Healthcare Digitalization: A Design Ethnographic Study
Source: Int J Public Health. 2024 Sep 6;69:1607575. doi: 10.3389/ijph.2024.1607575 (PMC11414544; doi:10.3389/ijph.2024.1607575)
Supplement: Supplementary file 2 [file DataSheet1.docx]

**Supplement Interview guidelines: design ethnographic use cases**

Participant codes are subsequently added by the data protection officer.

Date: _______________

Each participant may only be assigned a participant code once for the entire project (see pseudonymization list).

**Intro and input: approx. 5 minutes**

Welcome and introduction of the researcher ________________

Overall process of design ethnographic approach:

1. Visit (1. Usual usage of digital technology, think aloud & interview), 2. Feedback loop usual usage and idealization of digital technology with the help of co-creative method (interview with storyboarding and process mapping), 3. Feedback loop using the co-creative method (interview with storyboarding and process mapping)

Instructions: wearing a mask; reference is made to the voice recorder; asking for consent to voice record; verbal consent is requested again before the interview begins.

**Detailed explanation 1. Visit**

- Duration approx. 30 min; survey of the usage of digital technology and the associated perceived safety; qualitative content analysis of the data and the associated materials, knowledge, and needs; transformation into a storyboard and process mapping.
- Explanation Think Aloud method: the digital technology is used/carried out as usual, including preparation and follow-up actions; each step of the usage is explained aloud and the thoughts on it, the things necessary for usage are named if possible and the individual steps are justified if possible.
- Start voice recording/double recording.
- Participants‘ characteristics were collected

| **VISIT 1** | | |
| --- | --- | --- |
| **Conversation prompt** | **(Follow-up) questions** | **Instructions** |
| **Usual usage of digital technology** | | **Approx. 10-20 minutes** |
| Context Think Aloud   1. Please start with the usage of digital technology and tell the respective steps and thoughts out loud. | 1. **Daily usage**   **Show variant of usage (e.g. emergency)** | Interviewers make their own notes for the second part (under section "notes for moderators") on the aspects that the participants mention in order to be able to establish a connection (to perceived safety) |
| **Perceived safety** | | **Approx. 15 minutes** |
| **To what extent is what you have reported regarding digital technology, is related to the aspect of feeling safe?** | - What does this mean for your perceived safety? - What do you associate with the feeling you mentioned?   Questions about material, skills and meaning | - Paraphrasing initial thoughts - Inquire whether understood correctly - The points mentioned in Part 1 should be picked up on and placed in the context of feeling safe. - If necessary: Guidance on the perceived safety and aspects related to perceived safety. - Note: which aspects are related to feeling safe. - Sketching: important details are sketched out |
| **Further** | | **Approx. 5 minutes** |
| **When you think about our conversation, what else would be important to you in conclusion?** |  |  |
| **Thanking participants (**Reference to second and third visit) | |  |

**Detailed explanation 2nd visit**

- Duration approx. 45 min; validation of the current situation (feedback loop) with the help of the storyboard/process mapping; idealization of the process/usual usage with the help of the storyboard/process mapping; analysis of idealization and the associated materials, knowledge, and needs; preparation in a storyboard and process presentation
- Explanation of users as experts: "Nobody knows the usage of digital technology and the feelings it triggers in you as well as you do."
- Explanation Value-free idea space; every idea is an important idea and should be expressed.
- Start voice recording/double recording.

| **VISIT 2** | | |
| --- | --- | --- |
| **Conversation prompt** | **(Follow-up) questions** | **Instructions** |
| **Feedback loop / validation of usual usage of digital technology** | | **Approx. 10-15 minutes** |
| 1. **Explanation of the usual procedure /usage of DT** 2. **Would you like changes to the presentation of the usage process and the digital technology (incl. variant)?** | Does something need to be changed?  If applicable: "To what extent are there moments in the usual usage that have to do with feeling safe? | - Sketches are presented - Interviewers take notes themselves for the second part (under notes for moderators) on the aspects that the participants mention in order to be able to establish a connection (to perceived safety) |
| **Idealization** | | **Approx. 25 minutes** |
| **What is your ideal process and the associated usage of DT - in terms of your perceived safety?** | - What does this mean for your perceived safety? - What do you associate with the feeling you mentioned?   Questions about material, skills and meaning | - Outline initial thoughts and changes - Inquire if correctly understood/presented - The points mentioned in part 1 should be taken up and placed in the context of feeling safe. - If necessary: Guidance towards the perceived safety and aspects related to it. - Guiding people to have the courage to mention the "impossible" - Note: which aspects are related to the perceived safety - Sketching: important details will be sketched |
| **Conclusion** | | **Approx. 5 minutes** |
| **When you think about our conversation, what else would be important to you in conclusion?** | (Question about prioritization of idealization) |  |
| **Expression of thanks**   - Reference to last conversation | |  |

**Detailed explanation 3. Visit**

- Duration approx. 30 min; validation of the idealization (feedback loop); wishes for the future; preparation in a storyboard and process mapping.
- Explanation of users as experts: "Nobody knows the usage of digital technology and the feelings it triggers in you as well as you do."
- Explanation Value-free idea space; every idea is an important idea and should be expressed.
- Start voice recording/double recording.

| **VISIT 3** | | |
| --- | --- | --- |
| **Conversation prompt** | **(Follow-up) questions** | **Instructions** |
| **Feedback loop / validation of the idealization** | | **Approx. 5 minutes** |
| **Explanation of the ideal process / ideal usage** |  | - Sketches are presented |
| **Correction/adjustment of the idealization** | | **Approx. 10 minutes** |
| **Do you wish to make changes to the presentation of your ideal usage process and digital technology (incl. variant)?** | - What does this mean for your perceived safety? - What do you associate with the feeling you mentioned?   Questions about material, skills and meaning | - Outline initial thoughts and changes - Inquire if correctly understood/presented - The points mentioned in part 1 should be taken up and placed in the context of feeling safe. - If necessary: Guidance towards the perceived safety and aspects related to it. - Guiding people to have the courage to mention the "impossible" - Note: which aspects are related to the perceived safety - Sketching: important details will be sketched |
| **Conclusion** | | **Approx. 5 minutes** |
| **What would you like to see in the future with regard to perceived safety and digital technologies?** | (Question about prioritization of idealization) |  |
| **Expression of thanks and wrap up**   - Reference to the innovative character of the idealized application | |  |

**Moderator's notes for each visit:**

**1. Visit: Status quo, perceived safety, conclusion**

**2. Visit: Feedback loop / validation of status quo, perceived safety, idealization, conclusion**

**3. Visit: Feedback loop / validation of the idealization, correction/adjustment of the idealization, conclusion**
